# Interaction effects between smoking and internet gaming disorder on resting-state functional connectivity of the ventral tegmental area and hippocampus

**DOI:** 10.3389/fnins.2023.1270014

**Published:** 2023-10-25

**Authors:** Xianxin Qiu, Xu Han, Yao Wang, Weina Ding, Yawen Sun, Hao Lei, Yan Zhou, Fuchun Lin

**Affiliations:** ^1^Third-Grade Pharmacological Laboratory on Traditional Chinese Medicine, State Administration of Traditional Chinese Medicine, China Three Gorges University, Yichang, China; ^2^College of Medicine and Health Sciences, China Three Gorges University, Yichang, China; ^3^National Center for Magnetic Resonance in Wuhan, State Key Laboratory of Magnetic Resonance and Atomic and Molecular Physics, Wuhan Institute of Physics and Mathematics, Innovation Academy for Precision Measurement Science and Technology, Chinese Academy of Sciences, Wuhan, China; ^4^Department of Radiology, Ren Ji Hospital, School of Medicine, Shanghai Jiao Tong University, Shanghai, China; ^5^University of Chinese Academy of Sciences, Beijing, China

**Keywords:** internet gaming disorder, smoking, interaction, functional connectivity, ventral tegmental area, hippocampus

## Abstract

**Background:**

Many reports have focused on cigarette smoking and internet gaming disorder (IGD), with widespread alterations of resting-state functional connectivity (rsFC) in the reward and memory circuits, respectively. Epidemiological studies have also shown high comorbidity of cigarette smoking and IGD. However, the underlying mechanisms are still unknown. Therefore, this study investigates the comorbidity and interaction effects between smoking and IGD from the rsFC perspective.

**Methods:**

Resting-state functional magnetic imaging data were collected from 60 healthy controls (HC), 46 smokers, 38 IGD individuals, and 34 IGD comorbid with smoking (IGDsm) participants. Voxel-wise rsFC maps were calculated for all subjects with the ventral tegmental area, rostral hippocampus, and caudal hippocampus as regions of interest, respectively.

**Results:**

Significant interaction effects between smoking and IGD were mainly involved in the reward and memory circuits; that is, the rsFC between the ventral tegmental area and right nucleus accumbens, between the rostral hippocampus and bilateral nucleus accumbens, sensorimotor areas, and left middle temporal gyrus. Specifically, in these circuits, smokers showed decreased rsFC compared to the HC group, while IGDsm showed increased rsFC compared to smokers and IGD individuals. The IGDsm and HC groups showed no significant difference. The altered rsFC also correlated with clinical measures.

**Conclusion:**

These findings indicate that lower rsFC in smokers or IGD individuals increases under the effect of another type of addiction, such as smoking and IGD, but only increases to the normal state, which might explain the comorbidity and interaction between smoking and IGD from the perspective of functional circuits.

## 1. Introduction

Accumulating evidence showed that cigarette smoking is associated with respiratory and vascular diseases and even increases the risk of cancer, especially lung cancer (Doll and Hill, 1956). There is a higher mortality due to lung cancer in smokers than in non-smokers and in heavy smokers than in light smokers (Doll and Hill, 1956). It has been affirmed around the world that smoking cessation could significantly improve quality of life (Goldenberg et al., 2014). Internet gaming disorder (IGD) is often an outcome of the persistent and recurrent use of the Internet to engage in games (American Psychological Association [APA], 2013). IGD may lead to job loss and school and marriage failure (Picciotto et al., 2002). Many epidemiological reports demonstrate that cigarette smoking is associated with IGD, and smoking could be an important predictor of youth initiation and of the persistence of IGD (Chang et al., 2014; Dib et al., 2021).

Smoking and IGD are typical representatives of substance and behavior addiction and many studies have found that the brain changes caused by smoking and IGD both involve the ventral tegmental area (VTA) and the hippocampus. For example, structurally, smokers showed significantly smaller volumes, as well as greater volume loss with increasing age than non-smokers in the bilateral total hippocampus and multiple subfields (Durazzo et al., 2013). Individuals with IGD showed higher volume in the hippocampus than controls, and the volume positively correlated with the symptom severity of IGD (Yoon et al., 2017). Functionally, smokers showed greater activity after exposure to smoking-related images than after exposure to neutral images in the VTA and posterior hippocampus, while non-smokers showed no significant differences (Due et al., 2002). The resting-state functional connectivity (rsFC) between the hippocampus and striatum was higher during memory reconsolidation in the propranolol group (Lin et al., 2021). Long-term smokers had weaker effective connectivity from the VTA to the left ventral striatum (Zhang et al., 2022). In comparison with controls, both smokers and individuals with IGD had significantly decreased rsFC between the VTA and right nucleus accumbens (Zhang et al., 2015; Shen et al., 2016). These findings may suggest that the VTA and the hippocampus play an important role in brain function alteration in smokers and IGD individuals.

The VTA is the starting brain region of the reward dopamine mesolimbic circuit (Haber and Knutson, 2010), which is involved in processing emotional, motivational, and social reward associations (Douma and De Kloet, 2020). Addiction appears to be associated with a state of low dopaminergic dysfunction in the bran’s reward circuits (Gardner, 2011) and abnormalities in hippocampal-mediated memory and negative emotional responses (Tropea et al., 2008; Fanselow and Dong, 2010; Gardner, 2011; Domi et al., 2019). Many studies also found that smoking and IGD might share similar neural mechanisms in terms of brain activities and rsFC (Ko et al., 2009, 2013; Ge et al., 2017). A machine learning model based on rsFC also suggests that tobacco use disorder and IGD might share common neurological patterns (Chen et al., 2023). However, there have been no studies conducted on the rsFC of smoking and IGD comorbidities and the interaction effects between smoking and IGD in reward and memory circuits. Our previous studies found significant interaction effects between smoking and IGD on spontaneous brain activity and functional brain networks (Qiu et al., 2020, 2022). Therefore, we assume that there may also be interaction effects between smoking and IGD on rsFC of the VTA and hippocampus.

To test our hypothesis, we recruited four groups of healthy controls (HC), smokers, IGD, and IGD comorbid with smoking (IGDsm) individuals using resting-state functional magnetic resonance imaging (rs-fMRI) to investigate the rsFC alterations among addicts and the interaction effects of rsFC between smoking and IGD in reward and memory circuits with the VTA, rostral hippocampus (rHip), and caudal hippocampus (cHip) as the regions of interest (ROIs).

## 2. Materials and methods

### 2.1. Participants and measures

A total of 62 HCs, 50 smokers, 39 IGD subjects, and 34 IGDsm subjects were recruited in our study (age: 16–29 years). The Mini International Neuropsychiatric Interview (Sheehan et al., 1998) was used to exclude patients with a history of drug abuse or dependence, psychiatric or neurological diseases, or intellectual disability, excluding smokers and IGD individuals. Smokers met the criteria of tobacco use disorder in the fifth edition of the Diagnostic and Statistical Manual of Mental Disorders (American Psychological Association [APA], 2013) and smoked at least 10 cigarettes per day without quitting for more than 3 months during the last year. Smoking severity and craving were evaluated using the Fagerström test for nicotine dependence (FTND) (Heatherton et al., 1991) and the Questionnaire of Smoking Urges (Cox et al., 2001), respectively. Non-smokers in our study had smoked no more than five cigarettes during their lifetime. Smokers were not in acute withdrawal and approximately 1 h had passed since their last cigarette before MRI scanning. The IGD participants, recruited from the Shanghai Mental Health Center, met the criteria of modified Young’s Diagnostic Questionnaire for Internet Addiction (Beard and Wolf, 2001). These IGD participants also met the criteria of the fifth edition of the Diagnostic and Statistical Manual of Mental Disorders (American Psychological Association [APA], 2013) and there was no requirement that IGD participants could not play internet games the day before MRI scanning nor that they were not in acute withdrawal. The severity of IGD was assessed with the Chen Internet Addiction Scale (CIAS) (Ko et al., 2005). In addition, information was collected from all the participants using the Self-rating Anxiety Scale (SAS) (Zung, 1971), Self-rating Depression Scale (SDS) (Zung, 1965), and Barratt Impulsiveness Scale (BIS) (Patton et al., 1995).

The study procedures were carried out in accordance with the Declaration of Helsinki and were approved by the Medical Ethics Committee of Ren Ji Hospital, School of Medicine of Shanghai Jiao Tong University. All subjects were informed about the study and all provided informed consent.

### 2.2. Image acquisition and data pre-processing

See Supplementary data for the details of the image acquisition and data preprocessing. Two HCs, one IGD subject, and four smoking subjects did not pass the head-motion exclusion criteria for subjects (displacement greater than 2 mm in the x, y, or z direction or head rotation greater than 2° or average frame displacement greater than 0.2 mm) and the remaining 178 participants were included in a subsequent rsFC analysis.

### 2.3. Regions of interest and functional connectivity

**FIGURE 1:**
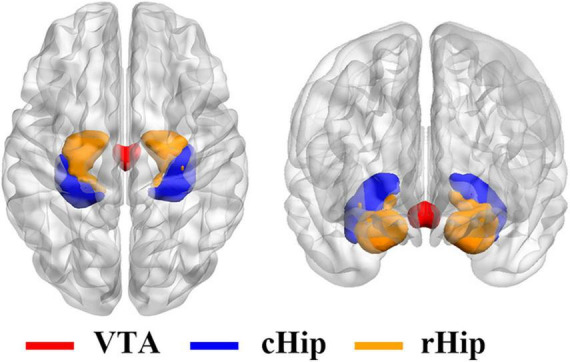
Three regions of interest for resting-state functional connectivity analysis. VTA, ventral tegmental area; cHip, caudal hippocampus; rHip, rostral hippocampus.

Considering the reward and memory circuits involved in smoking and IGD, the VTA and hippocampus were selected as ROIs (Fan et al., 2016; Faria et al., 2020). In addition, the posterior hippocampus performs primarily episodic memory and spatial navigation during cue-triggered relapse of addiction, while the anterior relates to stress, emotion, and affect during withdrawal (Fanselow and Dong, 2010; Gardner, 2011; Domi et al., 2019), so we divided the hippocampus into two ROIs, the rostral hippocampus and caudal hippocampus, referring to the Human Brainnetome Atlas (Fan et al., 2016). The brain ROIs are shown in Figure 1. For each ROI, the mean time series were estimated by averaging the signals of all voxels within that ROI. For each subject, the Pearson correlation coefficients of time series were calculated between each voxel and each ROI, followed by a Fisher’s r-to-z transformation to improve normality. Therefore, a functional connectivity map was constructed for each subject at each ROI.

### 2.4. Statistical analysis

To test demographic and clinical group differences, the Chi-square test was performed for the gender data and the two-way analysis of variance for other variables. As part of the following rsFC analysis, age, gender, years of education, and mean frame-wise displacement were used as the controlling variables and the statistical procedure included a voxel-wise one-sample *t*-test on rsFC maps with significant results saved as masks for the further two-way analysis of (ANCOVA) to test the interaction between smoking and IGD, both with a primary voxel determining threshold of *p* < 0.001 and FWE cluster-corrected (*p* < 0.05) to protect against false-positive findings. After that, the rsFC value of the significant cluster was extracted by averaging the rsFC values of all the voxels in the cluster and then performing one-way ANCOVA and *post hoc* comparison with Bonferroni’s correction (*p* < 0.05/5 = 0.01). To test the relationship between the altered rsFC and smoking as well as IGD-associated characteristics, a step-wise multiple linear regression analysis was performed with rsFC among groups as dependent variables, demographic features (i.e., age, education level, gender, and mean frame-wise displacement), smoking-related variables (i.e., duration of smoking, age at first smoking, and FTND), CIAS and other questionnaire scores (i.e., SAS, SDS, and BIS scores) as independent variables. A *p*-value of less than 0.01 was considered significant (uncorrected).

## 3. Results

### 3.1. Demographic and clinical measures

The detailed demographic and clinical information were the same as in our previous study (Qiu et al., 2020) and are listed in Supplementary Table 1.

### 3.2. One-sample *t*-test

**FIGURE 2:**
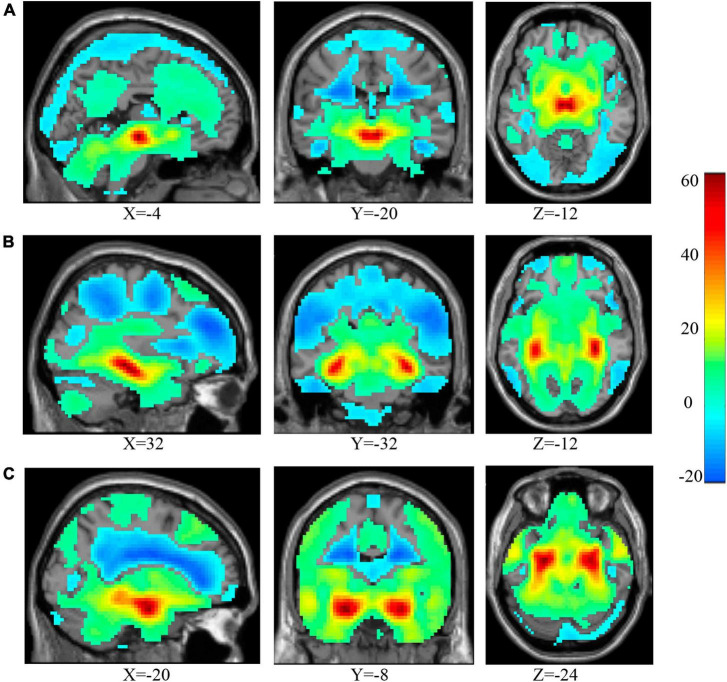
The results of one-sample *t*-test for voxel-wise rsFC between the three ROIs and the whole brain. As the VTA was the ROI **(A)**, the significant brain areas mainly involved the majority of the frontal gyrus, the temporal gyrus, parietal gyrus, cingulate gyrus, insula, middle brain, cerebellum, subcortical brain regions, and middle occipital gyrus. When the cHip was the ROI **(B)**, the significant brain areas mainly located in the frontal gyrus, temporal gyrus, cingulate gyrus, insula, the majority of the parietal gyrus, the middle occipital gyrus, and part of the subcortical brain regions. When the rHip was the ROI **(C)**, the significant brain areas are mainly located in the frontal gyrus, temporal gyrus, cingulate gyrus, insula, middle brain, brainstem, subcortical brain regions, the majority of the parietal gyrus and middle occipital gyrus. ROI, region of interest; VTA, ventral tegmental area; cHip, caudal hippocampus; rHip, rostral hippocampus.

The rsFC between the whole brain and three ROIs of each subject were calculated, respectively, and then a one-sample *t*-test was performed. The one-sample *t*-test results can be seen in Figure 2. As the VTA was the ROI (Figure 2A), the significant brain areas mainly involved the majority of the frontal gyrus, the temporal gyrus, parietal gyrus, cingulate gyrus, insula, middle brain, cerebellum, subcortical brain regions, and middle occipital gyrus. When the cHip was the ROI (Figure 2B), the significant brain areas mainly located in the frontal gyrus, temporal gyrus, cingulate gyrus, insula, the majority of the parietal gyrus, the middle occipital gyrus, and part of the subcortical brain regions. When the rHip was the ROI (Figure 2C), the significant brain areas were mainly located in the frontal gyrus, temporal gyrus, cingulate gyrus, insula, middle brain, brainstem, subcortical brain regions, the majority of the parietal gyrus, and the middle occipital gyrus.

### 3.3. Interaction effects of rsFC between smoking and IGD

**FIGURE 3:**
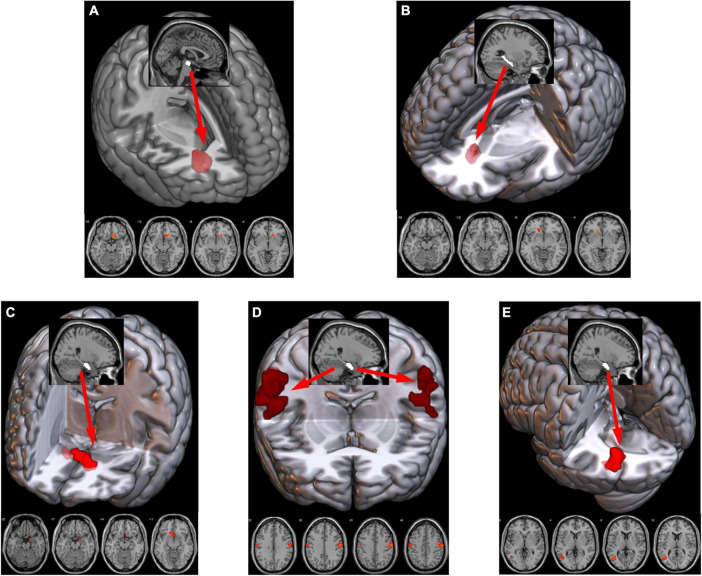
Significant interaction effects of rsFC between smoking and IGD were found between the VTA and right NAc **(A)**, between the cHip and left NAc **(B)**, between the rHip and bilateral NAc **(C)** bilateral SM, mainly involved in the post-central/pre-central gyrus **(D)** and the lMTG **(E)**. The sagittal view showed the ROI (white) and the axial view showed the clusters (red) with the rsFC interaction effects with the ROI. rsFC, resting-state functional connectivity; IGD, internet gaming disorder; VTA, ventral tegmental area; NAc, nucleus accumbens; cHip, caudal hippocampus; rHip, rostral hippocampus. The prefixes l and r stand for left and right, respectively. SM, sensorimotor area; MTG, middle temporal gyrus; ROI, region of interest.

After correction, the ANCOVA results showed that significant interaction effects between smoking and IGD were mainly involved in the rsFC between the VTA and the right NAc (Figure 3A) as well as between the rHip and the bilateral NAc (Figure 3C). The rsFC between the cHip and the left NAc (Figure 3B) did not pass the Bonferroni’s correction at the cluster level. In addition, interaction effects were also found between the rHip and the bilateral sensorimotor areas (SM, Figure 3D), mainly involved the posterior central gyrus and pre-central gyrus, as well as the left middle temporal gyrus (lMTG, Figure 3E). The peak coordinates, statistical *F*-values, and cluster sizes can be seen in Table 1.

**TABLE 1:** Clusters with significant interaction effects.

ROI	Brain region	Peak coordinates (MNI)			Location	*F*-value	Cluster size
		X	Y	Z			
VTA	Nucleus accumbens	9	21	−12	Right	16.84	72
cHip	Nucleus accumbens	−12	27	−6	Left	19.56	32
rHip	Nucleus accumbens	0	12	−9	Bilateral	19.53	82
	Sensorimotor area	63	−15	45	Right	24.60	203
	Sensorimotor area	−57	−12	48	Left	19.28	97
	Middle temporal gyrus	−39	−63	6	Left	18.50	95

**FIGURE 4:**
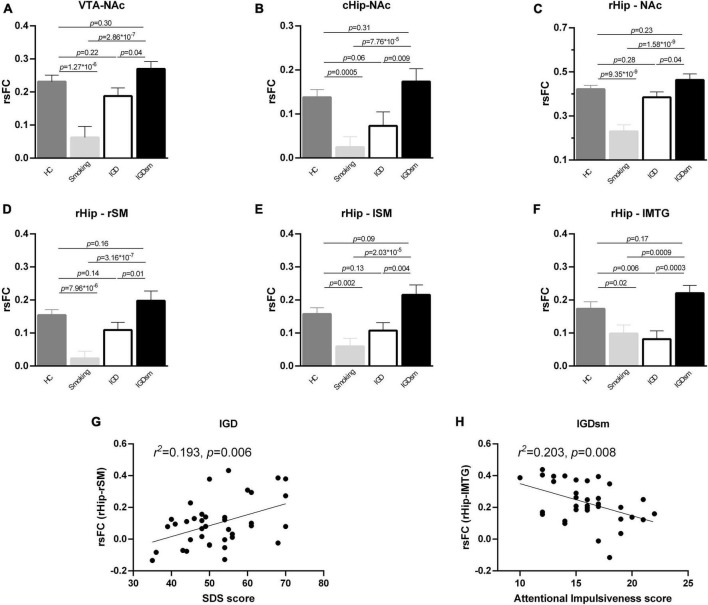
*Post hoc* analysis of rsFC between the selected ROIs and brain regions with significant interaction effects among groups **(A–F)** and correlations between the altered rsFC and clinical measures **(G,H)**. The smoking group showed significantly decreased rsFC compared to the HC group, while the IGDsm group showed significantly increased rsFC compared to the smoking group **(A–F)**. Furthermore, as for the rsFC between the cHip and the NAc **(B)** as well as between the rHip and lSM **(E)**, the IGD group showed no significant difference compared to the HC group, while IGDsm showed increased rsFC compared to the IGD group. As for the rsFC between the rHip and lMTG **(F)**, the IGD group showed significantly decreased rsFC compared to the HC group, while IGDsm showed significantly increased rsFC compared to the IGD group. The IGDsm group showed no significantly different rsFC compared to the HC group. **(G)** The rsFC between the rHip and rSM in the IGD group could be predicted by the SDS score (34 subjects, standardized β = 0.44, *t* = 2.936, *p* = 0.006). **(H)** The rsFC between the rHip and lMTG in the IGDsm group could be predicted by the Attentional Impulsiveness score (38 subjects, standardized β = –0.45, *t* = –2.853, *p* = 0.008). rsFC, resting-state functional connectivity; ROIs, regions of interest; HC, healthy controls; IGD, internet gaming disorder; IGDsm, IGD comorbid with smoking; VTA, ventral tegmental area; NAc, nucleus accumbens; cHip, caudal hippocampus; rHip, rostral hippocampus. The prefixes l and r stand for left and right, respectively. SM, sensorimotor area; MTG, middle temporal gyrus; SDS, self-rating depression scale.

*Post hoc* comparison of rsFC values among the four groups also showed significant results when the VTA, cHip, and rHip were used as the ROIs. Specifically, when the VTA was the ROI (Figure 4A), the interaction effects between smoking and IGD were significant [*F*_(1,174)_ = 22.67, *p* = 4.04 × 10^–6^]. The rsFC of smokers was significantly lower than that of the HC group (*p* = 1.27 × 10^–6^), but the rsFC of the IGDsm group was higher than that of smokers (*p* = 2.86 × 10^–7^). No significant difference was found between the IGD and HC groups (*p* = 0.22), while the rsFC of IGDsm was higher than that of IGD individuals, but it did not pass the Bonferroni’s correction (*p* = 0.04). No significantly different rsFC was found between the IGDsm and HC groups (*p* = 0.30).

Although the rsFC between the cHip and the left NAc did not pass the Bonferroni’s correction at the cluster level, we still extracted the cluster and performed one-way ANCOVA [interaction results between smoking and IGD: *F*_(1,174)_ = 18.37, *p* = 3.01 × 10^–5^] and *post hoc* comparison among groups (Figure 4B). The rsFC of smokers was significantly lower than that of the HC group (*p* = 0.0005), but the rsFC of the IGDsm group was significantly higher than that of smokers (*p* = 7.76 × 10^–5^). No significantly different rsFC was found between the IGD and HC groups (*p* = 0.06), but the rsFC of the IGDsm group was significantly higher than that of the IGD group (*p* = 0.009). No significant difference in rsFC was found between the IGDsm and HC groups (*p* = 0.31).

When the rHip was the ROI, the interaction effects between smoking and IGD were significant [*F*_(1,174)_ = 29.58, *p* = 1.80 × 10^–7^ for the NAc, *F*_(1,174)_ = 24.31, *p* = 1.90 × 10^–6^ for the rSM, *F*_(1,174)_ = 18.18, *p* = 3.29 × 10^–5^ for the lSM and *F*_(1,174)_ = 19.11, *p* = 2.12 × 10^–5^ for the lMTG, respectively]. The significant results of rsFC between the rHip, NAc, and the rSM were the same as between VTA and NAc, and the significant results of rsFC between the rHip and lSM were the same as between the cHip and NAc, except for the specific *p*-values, which can be seen in Figures 4C–E. As for the rsFC, between the rHip and lMTG (Figure 4F), no significant difference was found between smokers and the HC group (*p* = 0.02, did not passed the Bonferroni’s correction), but the rsFC of the IGDsm group was significantly higher than that of smokers (*p* = 0.0009). The rsFC of the IGD group was significantly lower than that of the HC group (*p* = 0.006), but the rsFC of the IGDsm group was significantly higher than that of the IGD group (*p* = 0.0003). No significantly different rsFC was found between the IGDsm and HC groups (*p* = 0.17).

### 3.4. Correlations between altered rsFC and clinical characteristics

The stepwise multiple linear regression analysis found that the rsFC between the rHip and rSM in the IGD group could be predicted by the SDS score (38 subjects, standardized β = 0.44, *t* = 2.936, *p* = 0.006, Figure 4G) and the rsFC between the rHip and lMTG in the IGDsm group could be predicted using the Attentional Impulsiveness score (34 subjects, standardized β = −0.45, *t* = −2.853, *p* = 0.008, Figure 4H). No other significant correlation was found between the altered rsFC and IGD or smoking-related characteristics.

## 4. Discussion

This study presents strong evidence about smoking and IGD comorbidity and their interaction effects on rsFC. The significant interaction effects between smoking and IGD, mainly involving in the rsFC between the VTA and the right NAc, between the rHip and NAc, sensorimotor area, and lMTG, were marked by decreased rsFC in smoking or IGD participants but increased rsFC in the IGDsm group under the influence of IGD or smoking. These results suggest that smoking has a higher comorbidities and interactions with IGD from the perspective of rsFC. We also found that the rsFC between the rHip and rSM could be predicted by the SDS score in the IGD group and the rsFC between the rHip and lMTG could be predicted by the Attentional Impulsiveness score in the IGDsm group.

We found significantly decreased rsFC between the right NAc and VTA in smokers, which was consistent with other reports of widespread rsFC attenuation in the reward circuit of smokers compared with non-smokers (Shen et al., 2016). IGD individuals also showed significantly decreased rsFC between the VTA and NAc and negative relationships between rsFC and cravings as well as the Internet Addiction Test (Zhang et al., 2015; Wang et al., 2019). We found no significantly decreased rsFC between the VTA and NAc and its associations with clinical measures in IGD individuals might be caused by the small samples. The essence of addiction is reward, and the VTA-NAc dopamine reward circuit appears to be at the center (Haber and Knutson, 2010). The addiction cycle can be conceptualized as three major components: preoccupation/anticipation, binge/intoxication, and withdrawal/negative affect (Koob, 2017). In the initial stage, impulsive addiction behaviors are often accompanied by feelings of pleasure or gratification (Koob and Volkow, 2016) and drug abuse could increase extracellular dopamine concentrations, especially in the accumbens, eliciting hyper-motility at low doses (Di Chiara and Imperato, 1988). The reward effect brought by acute addiction leads to a series of neurophysiological alterations, which may break the homeostasis (Koob, 1997). Therefore, in order to maintain the homeostasis of the internal environment, two possible mechanisms can be hypothesized to regulate the reward effect, mainly including a within-systems and a between-systems adaptive regulation (Koob and Bloom, 1988). In the within-systems opposing process, the primary cellular response element to the drug would itself adapt to neutralize the drug’s effects (Koob, 1997). For example, the opposing process of chronic cocaine on the reward effect is to increase the reward threshold and the magnitude, and the duration of the threshold is proportional to the amount of administrated cocaine (Markou and Koob, 1991). In addition, the increased threshold will not return to the baseline and it becomes further away from the baseline as the drug is used (Jang et al., 2013). This means that the initial “high” feeling caused by smoking or IGD cannot be maintained but rather decreases, which might explain the decreased rsFC between the VTA and NAc in smokers and IGD individuals.

The between-systems adaptive regulation of anti-reward mainly involves negative reinforcement and negative emotions, which are activated by the reward effect of substance or addictive behavior at the same time or after the reward effect occurs and inhibits the activated reward circuit together with within-systems regulation (Koob, 2017). Specifically, if the subjects do not smoke or play online games, they will have negative emotions, which refers to the anti-reward regulation that drives addicts to compulsively smoke or play online games to reduce this negative tension or anxiety (Koob and Volkow, 2010, 2016). In this context, individuals move from impulsivity to compulsivity, and the drive for behavior is paralleled by shifts from positive to negative reinforcement (Koob, 2017). However, because within-systems regulation raises the reward threshold, addicts increasingly need to consume more tobacco or play more online games to overcome these negative feelings. In other words, the reward effect of the same amount of stimulus becomes lower and lower. For example, the striatal increases in DA induced by intravenous methylphenidate or amphetamine in cocaine abusers and alcoholics are at least 50% lower than in control subjects and might reflect decreased DA cell activity likely in drug abusers (Volkow et al., 2009). This decreased sensitivity, on the other hand, would result in a reduced interest in environmental stimuli, possibly predisposing subjects to seeking drug stimulation as a means to temporarily activate these reward circuits (Volkow et al., 2009). As time progresses, the chronic nature of this behavior may explain the transition from taking drugs in order to feel “high” to taking them just to feel normal (Volkow et al., 2009). Nevertheless, although both the levels of D2 receptors and DA released in the striatum are reduced in abusers (Volkow et al., 2002), drugs are much more potent at stimulating DA-regulated reward circuits than natural reinforcers, and drugs are still able to activate the depressed reward circuits (Volkow et al., 2009). This could cause the addict to become stuck on the path to addiction and even increasingly need new drugs or addictive behaviors to seek to return to a “normal” state, which might explain the comorbidity of smoking and IGD and the increased rsFC between the VTA and NAc in the IGDsm group compared to the smoking or IGD group, while no significant difference was observed in the HC group.

We found decreased rsFC between the hippocampus (rHip and cHip) and NAc in smokers. The cHip is an important substrate for nicotinic effects on working memory function (Levin and Rezvani, 2002; Fanselow and Dong, 2010). The associations between rewarding properties of addictions and memory can contribute to future use and facilitate the transition from initial drug use into drug dependency (Kutlu and Gould, 2016). For example, the disruption of memory reconsolidation by propranolol increased rsFC between the hippocampus and striatum, and the increase in rsFC was correlated with the decrease in craving after the reconsolidation manipulation in the propranolol group (Lin et al., 2021). The within-systems adaptation of anti-reward through increasing the reward threshold and decreasing the DA and its D2 receptor was associated with tonic DA cell firing, which is achieved by activating the pathway of the ventral subiculum region of the hippocampus-NAc-ventral globus pallidus-VTA (Floresco et al., 2003). On the other hand, the rHip is involved in the anti-reward between-systems regulation of negative emotions (Fanselow and Dong, 2010), which could reduce the threshold for long-term potentiation between cHip and NAc (Yamazaki et al., 2006). This might suggest decreased rsFC between the hippocampus and NAc in smokers.

Repetitive drug administration can be associated with progressively increasing effects on drugs, that is, drug sensitization (Post and Rose, 1976), which is achieved through long-term potentiation mediated by D1 receptor activation between the ventral subiculum of the hippocampus and NAc synapses (Goto and Grace, 2005). Drug sensitization might cause the comorbidity of several substances and behavioral addiction. For example, we found that the rsFC of the IGDsm group was higher than that of smokers or IGD individuals, which might explain the comorbidity of smoking and IGD from the perspective of the rsFC between the hippocampus and NAc. On the other hand, smoking cessation leads to the occurrence of physical and affective withdrawal symptoms, representing a major obstacle to quitting tobacco use. Hippocampal microinjections of pioglitazone reduced the expression of the physical signs of withdrawal (Domi et al., 2019). The hippocampus is also involved in the regulation of negative emotional stress responses, such as the release of adrenergic release from the locus coeruleus, a central site in response to stress, which activates hippocampal neurons (Loy et al., 1980). The chronic nature of this behavior may explain the transition from taking drugs in order to feel “high” to taking them just to feel normal (Volkow et al., 2009), which might explain our result of no significant difference between the IGDsm and HC groups in rsFC between the hippocampus and NAc.

In addition, we found significant interaction effects of rsFC between the rHip and the sensorimotor area as well as the lMTG, which may be a reminder that these brain regions are also involved in the regulation in addiction. It has been documented that compared only to the drug itself, the cues related to the action of drug use are more likely to activate the sensorimotor area and promote a range of learning and plasticity processes associated with the hippocampus (Tian et al., 2011; Zeng et al., 2018). These processes may mediate cravings and automatic drug-seeking behavior (Zeng et al., 2018). For example, smokers spontaneously represent the action of smoking when viewing others smoke (Wagner et al., 2011), the consequence of which may make it more difficult to abstain from addiction and even seek other addictions to make themselves feel normal, like the comorbidity of smoking and IGD in our study. The MTG may be involved in social cognition, semantics, and association memory (Leshinskaya and Thompson-Schill, 2020; Xu et al., 2020). Our functional findings might indicate adaptive alteration of MTG with long-term exposure to the drug and the formation of associative memories of drug-related cues. We found that the rsFC between the rHip and lMTG in the IGDsm group could be predicted by the Attentional Impulsiveness score, which is consistent with less activation of the MTG and making risky decisions more hastily in IGD than in HC individuals (Dong and Potenza, 2016), which may imply that IGD and smoking individuals are associated with reduced inhibitory control ability (Zsido et al., 2019).

There are several limitations to this study. First, the possible impacts of poorly matched age, gender, or education among the groups could not be eliminated completely, although they were incorporated into the statistical design as covariates. Second, gender differences were not investigated in this study. Men and women seem to have different neurophysiology and performance during the addictive procedure, which needs to be explored in further approaches. Third, although we limited the time to approximately 1 h between the last cigarette and MRI scanning in smokers, concentrations of carbon monoxide were not applied to quantitatively assess whether all subjects were in the same state, which might affect the results and requires further approaches to verify these results. Fourth, we failed to assess the number of cigarettes per day consumed by the smokers, and its relationship with rsFC, therefore, could not be elucidated in this study and will require further research. Fifth, while most subjects self-reported no or very little daily alcohol consumption, we could not have known if they were lying. Therefore, the possible effects of alcohol consumption on the interaction between smoking and IGD need to be studied in the future.

In conclusion, our study found significant interaction effects of rsFC between smoking and IGD, which specifically manifested as a decrease in rsFC in a single type of addiction, which may be related to the increase in the reward threshold in the reward circuit caused by chronic addiction or the negative reinforcement of stress regulation. Instead, increased rsFC in the comorbidity of IGDsm group compared to a single type of addiction group might be due to drugs being much more potent at stimulating DA-regulated reward circuits than natural reinforcers. The lack of difference in rsFC between the IGDsm and HC groups might be related to the transition from taking drugs in order to feel “high” to taking them just to feel normal. In summary, our findings provide valuable fMRI evidence for the principle of chronic addiction, relapse, and comorbidity of two types of addiction from the perspective of functional reward and memory circuits.

## Data availability statement

The original contributions presented in this study are included in the article/Supplementary material, further inquiries can be directed to the corresponding authors.

## Ethics statement

The studies involving humans were approved by the Medical Ethics Committee of Ren Ji Hospital, School of Medicine of Shanghai Jiao Tong University. The studies were conducted in accordance with the local legislation and institutional requirements. Written informed consent for participation in this study was provided by the participants’ legal guardians/next of kin. Written informed consent was obtained from the individual(s) for the publication of any potentially identifiable images or data included in this article.

## Author contributions

XQ: Formal analysis, Methodology, Validation, Visualization, Writing – original draft, Writing – review and editing. XH: Data curation, Writing – review and editing. YW: Data curation, Writing – review and editing. WD: Data curation, Writing – review and editing. YS: Data curation, Writing – review and editing. HL: Conceptualization, Writing – review and editing. YZ: Data curation, Funding acquisition, Writing – review and editing. FL: Conceptualization, Funding acquisition, Project administration, Supervision, Writing – review and editing.
