# Supplementary material for: Interaction effects between smoking and internet gaming disorder on resting-state functional connectivity of the ventral tegmental area and hippocampus
Source: Front Neurosci. 2023 Oct 25;17:1270014. doi: 10.3389/fnins.2023.1270014 (PMC10641398; doi:10.3389/fnins.2023.1270014)
Supplement: Supplementary file 1 [file Data_Sheet_1.docx]

**Supplementary material**

**Image acquisition**

All MRI data were acquired by a 3.0-Tesla GE Signa HDx (Milwaukee, WI, USA) scanner using a standard 8-channel head-coil. To minimize the head motion and scanner noise, foam pads and earplugs were used while scanning. All subjects underwent a 3D T1-weighted fast spoiled gradient echo sequence with the parameters: repetition time (TR)/echo time (TE) = 6.1/2.8 ms; inversion time = 450 ms; flip angle = 15°; matrix = 256×256; field of view (FOV) = 256×256 mm^2^; slice thickness = 1 mm with no gap and 156 sagittal slices. Rs-fMRI data were obtained using the echo-planar imaging sequence with the parameters: TR/TE = 2000/24 ms; flip angle = 90°; matrix = 64×64; FOV = 230×230 mm^2^; 4 mm without gap and 34 axial slices. The rs-fMRI scan lasted for 440 seconds, and 220 volumes were collected. All the images were visually evaluated by two experienced neuroradiologists to exclude the possible pathological findings.

**Data preprocessing**

Rs-fMRI data were preprocessed by the Data Processing Assistant for Resting-State fMRI (DPARSF) toolkit (Yan et al., 2016). The first 10 volumes for each subject were discarded for magnetic equilibrium, and the remaining volumes were performed with slice-timing and head-motion correction. After that, the individual 3D-T1 structural image was co-registered to the mean functional image and segmented into gray matter, white matter (WM), and cerebrospinal fluid (CSF). Finally, transformation from individual native space to Montreal Neurological Institute (MNI) space were acquired using the Diffeomorphic Anatomical Registration Through Exponentiated Lie algebra (DARTEL) (Ashburner, 2007). To minimize the head-motion confounds, the Friston 24-parameter model was used (Friston et al., 1996). To reduce respiratory and cardiac effects, WM and CSF signals were also regressed out (Behzadi et al., 2007). Global signal is not regressed since it is controversial practice. It yields subsequent observations of negative correlations (Murphy et al., 2009, Weissenbacher et al., 2009) and may distort group differences in intrinsic functional connectivity (Saad et al., 2012). Additionally, linear and quadratic trends were included as regressors to account for drifts in the blood oxygen level dependent signal. After these procedures, the functional images were normalized to the MNI space using DARTEL. Finally, the normalized functional images were temporally band-pass filtered (0.01-0.08 Hz) and smoothed with a 6 mm full width at half maximum Gaussian kernel.

**References**

Ashburner, J. (2007). A fast diffeomorphic image registration algorithm. *Neuroimage,* 38**,** 95-113. doi: 10.1016/j.neuroimage.2007.07.007

Behzadi, Y., Restom, K., Liau, J. and Liu, T. T. (2007). A component based noise correction method (CompCor) for BOLD and perfusion based fMRI. *Neuroimage,* 37**,** 90-101. doi: 10.1016/j.neuroimage.2007.04.042

Friston, K. J., Williams, S., Howard, R., Frackowiak, R. S. and Turner, R. (1996). Movement-related effects in fMRI time-series. *Magnetic Resonance in Medicine,* 35**,** 346-55. doi: 10.1002/mrm.1910350312

Murphy, K., Birn, R. M., Handwerker, D. A., Jones, T. B. and Bandettini, P. A. (2009). The impact of global signal regression on resting state correlations: are anti-correlated networks introduced? *Neuroimage,* 44**,** 893-905. doi: 10.1016/j.neuroimage.2008.09.036

Saad, Z. S., Gotts, S. J., Murphy, K., Chen, G., Jo, H. J., Martin, A., et al. (2012). Trouble at rest: how correlation patterns and group differences become distorted after global signal regression. *Brain Connect.,* 2**,** 25-32. doi: 10.1089/brain.2012.0080

Weissenbacher, A., Kasess, C., Gerstl, F., Lanzenberger, R., Moser, E. and Windischberger, C. (2009). Correlations and anticorrelations in resting-state functional connectivity MRI: a quantitative comparison of preprocessing strategies. *Neuroimage,* 47**,** 1408-16. doi: 10.1016/j.neuroimage.2009.05.005

Yan, C. G., Wang, X. D., Zuo, X. N. and Zang, Y. F. (2016). DPABI: Data Processing & Analysis for (Resting-State) Brain Imaging. *Neuroinform.,* 14**,** 339-51. doi: 10.1007/s12021-016-9299-4

**Table S1.** Demographic and clinical information for subjects in each group and comparisons of four groups.

|  | IGD group (n=38) | IGDSmoking group (n=34) | HC group (n=60) | Smoking group (n=46) | p value | | |
| --- | --- | --- | --- | --- | --- | --- | --- |
|  |  |  |  |  | IGD | Smoking | IGD * Smoking |
| Age | 19.66 ± 2.60 | 22.94 ± 2.93 | 22.29 ± 3.48 | 22.88 ± 2.78 | 0.006 | <0.001 | 0.004 |
| Gender (M/F) | 26//12 | 33//1 | 41//19 | 31//15 | - | - | 0.008^a^ |
| Education | 10.74 ± 1.75 | 10.29 ± 2.10 | 14.76 ± 3.39 | 11.83 ± 2.50 | <0.001 | 0.003 | 0.06 |
| Duration of smoking | - | 3.75 ± 1.88 | - | 5.18 ± 2.92 | - | - | - |
| Age at first smoking | - | 19.19 ± 2.83 | - | 17.70 ± 3.01 | - | - | - |
| FTND | - | 6.68 ± 2.07 | - | 6.28 ± 2.25 | - | - | - |
| CIAS score | 72.68 ± 10.27 | 81.26 ± 10.69 | 43.83 ± 10.83 | 49.93 ± 10.57 | <0.001 | <0.001 | 0.45 |
| SAS score | 48.89 ± 9.57 | 57.24 ± 11.56 | 40.53 ± 7.40 | 47.59 ± 9.93 | <0.001 | <0.001 | 0.66 |
| SDS score | 52.08 ± 9.41 | 58.00 ± 8.90 | 44.32 ± 8.62 | 50.65 ± 9.70 | <0.001 | <0.001 | 0.88 |
| BIS-11 score | 61.63 ± 8.19 | 64.12 ± 8.62 | 52.80 ± 6.91 | 55.67 ± 10.00 | <0.001 | 0.039 | 0.88 |
| BIS - Attentional Impulsiveness score | 15.16 ± 2.69 | 15.97 ± 2.90 | 12.98 ± 2.30 | 13.13 ± 2.80 | <0.001 | 0.24 | 0.41 |
| BIS - Motor Impulsiveness score | 20.63 ± 4.46 | 21.82 ± 3.64 | 17.92 ± 2.96 | 19.91 ± 4.00 | <0.001 | 0.006 | 0.48 |
| BIS - Nonplanning Impulsiveness score | 26.16 ± 3.48 | 26.50 ± 4.66 | 21.90 ± 4.47 | 22.63 ± 5.62 | <0.001 | 0.45 | 0.79 |

^a^ The Chi-square test demonstrated significant sex differences among the four groups (*p* = 0.008).

Note. Values are expressed as mean ± standard deviation. The age, educational level, age at first cigarette and duration of smoking are displayed in years. FTND: Fagerström Test of Nicotine Dependence; CIAS: Chen internet addiction scale. SAS: Self-rating Anxiety Scale; SDS: Self-rating Depression Scale; BIS-11; Barratt Impulsiveness Scale, version 11. Educational level was defined as the number of years of scholarship since primary school.
